# Repertory to Labor and after Pains

**Published:** 1889-07

**Authors:** John V. Allen

**Affiliations:** Philadelphia


### REPERTORY TO LABOR AND AFTER PAINS.

JOHN V. ALLEN, M. D., PHILADELPHIA.

### LABOR-PAINS.

Abdomen, with cutting pains from before backward and upward, *in*—Gels.
" " " " in—Phos.
" " cramps in, and shooting down the legs—Viburnum.
" " weak feeling in—Phos., Sepia.

Air, must have fresh, cool—Pulsatilla, Cham.
Amniotic fluid gone, with—Bell.
Anguish, with—Nat. carb., Acon.
Appearing and disappearing suddenly—Bell.
Back, pains begin in, and pass off down the buttocks—Kali carb.
" go up to—Gels.
" cutting across lumbar region—Kali carb.
" worse in—Nux vom., Caust.
Bear, which she can hardly—Cham.
Belching, with relief by—Kali carb.
Body cool, with—Arn.
Bruised feeling in body, with—Arn.
Breathing deeply, with—Ignatia.
Ceasing—Bell., Cham., Carb. veg., Cauloph., Cimicif., Gels., Graph., Kali c., Nat. mur., Nux-vom., Op., Plat., Puls., Ruta, Sepia, Secale., Sulph., Thuja.
" from hæmorrhage—China.
" " violent diseases—Carb. veg.
Cessation of (entire)—Cimicif., Guare.
Cervix, with needle-like pain in—Caul.
" " shooting in, extending upward—Sep.
Chest, go up to—Gels.
Cramps in lower extremities, with—Cuprum.
Contraction, hour-glass with—Bell., Cham.
Convulsions, with—China, Secale.
" hysterical interrupt the—Mag. mur.
Covered, will not be during—Camph., Secale.
Cutting—Gels., Ipec., Phos.
Darting—Ipec.
Distress, with great—Acon.
Distressing—Acon., Arn., Aurum., Bell., Cham., Cimicif., Coffea., Con., Gels., Kali c., Lyc., Nux-vom., Phos., Plat., Sep., Secale.
Death, with extreme fear of—Acon., Coffea.
Desperate, make her—Aurum.
Drowsiness, with—Nux-mos.
Dryness of parts, with—Acon.
Dyspnœa, with—Puls.
Endure, she cannot—Cham.
Eructations violent, with—Borax.
Eyes injected, with—Opium.
Exhaust her—Verat. alb.
" " she is out of breath—Stannum.

Exhaustion, from long protracted labor—Caul.
Face flushes up red, with—Arn., Ferrum.
" hot, with—Bell.
" dark red, with—Op.
Fainting, with—China, Cimicif., Ipec. Mag. mur., Nux-vom., Nux-mos., Puls., Secale.
Fainting, every pain causes—Nux-vom.
" from least motion, during—Verat. alb.
Fever, with—Caul.
Frantic, which render her—Cham.
Foot against a support, and pushing and relaxing alternately; relief by—Lyc.
Forebodings, with Nat. mur.
False—Caul., Nux-vom., Viburnum.
Grief—Caust.
Hands touched, cannot bear to have, during—China.
Hæmorrhage, with—China.
Hard one, with several light ones after long interval—Coccul.
Head hot, with—Arnica.
" congestion to, with—Aurum.
Headache throbbing, with—Bell., Coccul.
Heart, palpitation of, with—Aurum., Puls.
Ineffectual—Coffea, Plat., Puls.
Inefficient—Acon., Bell., Caust., Gossypium, Ustilago.
Insupportably—Coffea.
Interrupted—Platina.
Irregular—Æthusa cyn., Caul., Coccul., Cup., Nux-mos., Puls., Secale.
Irritability, with—Cham.
Jarring of bed, sensitive to—Bell.
Jerking, with—Opium.
Lamenting, with—Coffea.
Legs, with numb and paralyzed feeling in—Coccul.
" " shooting down the—Viburnum.
" " tearing down the—Cham.
Light, sensitive to, with—Bell.
Lingering, almost painless—Gossypium.
Located, not rightly—Cimicif.
Moaning, with—Acon.
Motion, must keep in constant—Lycop.
Nausea, with—Ipec., Mag. mur.
Needle-like—Sepia.
Noise, sensitive to, with—Bell., Cimicif.

Os uteri. Contraction, spasmodic, with—Bell.
" Dry, with—Acon., Bell.
" Dilated, but patient has become tired and fretful, with—Caust.
" Dilatable with—Ustilago.
" Hot, with—Bell.
" Rigidity, with—Caul., Cham., Cimicif., Conium., Gels.
" Soft, with—Ustilago.
Painful—Platina.
Pains similar to—Cham., Camph., Juni., Kreos.
Perspiration, with—Nat. carb.
Progress slowly—Caul., Nat. mur., Puls.
Prolonged and forcing—Secale.
Pulse weak, with—Secale.
Relaxed, everything seems, during—Secale.
Rapid succession, follow in—Acon.
Restlessness, with—Acon., Arn.
" " between pains—Cup.
Rubbed, desire to be, which relieves, during—Nat. carb.
Sacrum, with only slight pressure on—Bell.
Sluggish—Puls.
Severe—Cimicif., Coffea.
Sharp—Kali carb.
Short—Caul.
Shivers, with (in first stage)—Cimicif.
Shrieks, with—Cham.
Shuddering during, wants to be covered—Sepia.
Skin cold, with—Camph., Secale.
Sleeplessness, with—Mag. mur.
Sleepiness (drowsy) with—Nux-mos., Opium.
Slow—Bell., Con., Nux-mos.
Sopor, with—Op.
Soreness all over, with—Arn., Ruta.
Spitefulness, with—Cham.
Spasmodic—Caul., Cham., Cimicif., Caust., Coccul., Conium, Cup., Nux-vom., Nux-mos., Plat., Puls., Stannum.
Stool, with urging to—Nux-vom.
" retention of, with—Op.
Strong, too—Bell., Cham., Coff., Con., Nux-vom., Puls., Secale.
Sudden—Bell.
Suppressed—Nux-mos.
" from fear—Op.

Suppressed from fright—Op.
Tearing—Cimicif., Cham.
Tedious—Bell., Cimicif.
Tardy—Gels.
Tenderness of parts, with—Acon.
Thirst, with—Caul.
Touched, cannot bear to be, during—China.
Tumor, with—Nat. carb.
Twitching, with—Op.
Undilatable parts, with—Acon.
Umbilicus, cutting about, darting toward uterus—Ipec.
Upward, go—Lyc.
Urinate, with urging to—Nux-vom.
Urine, with retention of—Op.
Vagina, dry, with—Acon., Bell.
" rigidity of, with—Ars.
" sensitiveness of, interrupt—Platina.
Vulva, dry, with—Acon.
Vertigo on turning in bed, with—Conium.
Violent, but do little good—Arnica, Caul., Coff., Phos., Plat.
Weeping, with—Coffea, Lyc., Nat-mur., Puls.
Window, with desire to jump out of—Aurum.
" " " " have open—Puls.
Weak, too—Arn., Æthusa, Bell., Cann., Caul., Cimicif., Gels., Kali carb., Opium, Puls., Secale; *Also*—Borax, Camph., Carb-veg., Cham., Cocc., Graph., Ign., Lyc., Mag-mur., Nat-mur., Nux-mos., Nux-vom., Plat., Ruta, Sep., Sulph., Thuja.
Weak, patient very—Caul.
Women, in corpulent—Graph.
" " tall, slender—Phos.
" " cachectic—Secale.
" " blonde—Viburnum.

### After-Pains.

Abdomen, spasmodic across lower, extends into groins—Caul.
" violent in—Ferrum.
" with sensitiveness of—Sabina.
Air, wants fresh—Cham.
Anus, with constant sense of weight in—Sepia.
Back, felt mostly in—Sepia.
" with bearing down or forcing in—Sep.
" stitching and shooting pains in, going down to gluteal region or hips—Kali carb.
Bearing down, with strong—Podoph.

Breathing, excited by—Bry.
Covered, aversion to being, though surface cold—Secale.
" relief by being—Rhus tox.
Cramping—Cuprum, Coloc.
" in calves—Rhus tox.
" " extremities, causing—Cup.
Delirium, with—Hyoscy.
Delivery, after instrumental—Hypericum.
Death, with extreme fear of—Acon., Coff.
Despondency, with—Ign.
Distressing—Cham., Coff., Cup-met.
Endure them, she cannot—Cham.
Evening, worse in—Puls.
Extending from left to right—Conium.
Face fiery red, with—Ferrum.
Faint, weak feelings, with—Sulph.
Feelings changeable, now better, now worse, with—Puls.
Fingers, causing cramp in—Cup.
Flatulency with—Podoph., Nux-mos.
Forcing, as if contents of pelvis would be forced through vulva—Bell.
Groins, extends to—Caul., Cimicif.
Headache agonizing, with sensation as though the face was drawn toward root of nose, then backward toward occiput, as if by a string; eyeballs painful and sore to the slightest attempt at motion—Paris quad.
Headache severe in right side back of orbit, with—Cimicif.,
" with—Ferrum.
Heat, with flashes of—Sepia, Sulph., Podoph.
Intense, with imperfect contractions of uterus—Paris quad.
Jerking, with—Hyos.
Long, too—Puls.
Lochia brown and thin—Secale.
" dark-colored, with—Cham.
Lochial discharge which seems hot, with—Bell.
" " increased with each pain—Bell., Xanthoxylum.
" " scanty—Sulph.
" " Suppression of, entire—Paris quad.
Labor-like, with discharge of partly fluid and partly clotted blood—Ferrum.
Loins, violent in—Ferrum.
Low-spiritedness, with—Cimicif.
Motion, excited by—Bry.

Motion, relief by—Rhus tox.
Multiparæ, in—Cuprum.
Nausea and vomiting, with—Cimicif.
Night, worse at, hardly any during the day—Rhus-tox.
Nursing excites—Conium, Arn.
Over-sensitiveness, with—Cimicif.
Painful, too—Acon.
Prolonged—Acon., Secale,
Protracted labor, after—Caul.
" too—Nux-vom.
Pubes, pains from sacrum to—Sabina.
Restlessness, with—Cimicif., Puls.
Sacrum and hips, with severe headache, violent in—Hypericum.
" to knees and ankles, thence up to sacrum, jerks here and there; from—Phyto.
Sadness, with—Ign.
Sensitiveness to the pains, with—Cimicif.
Severe, too, and too long lasting—Gels.
Shuddering, frequent, with—Ferrum.
Sighing, with much—Ign.
Sleep, cannot compose themselves to—Gels.
" prevented through sleepy.—Coffea.
Sleeplessness, with—Cimicif.
Soreness in uterine region, so that she dreads to be disturbed, with—Nux-vom.
Spasmodic—Hyos.
" across lower abdomen—Caul.
Stool, with desire for, with every pain—Nux-vom., Paris quad.
Tenderness to pressure, uterus does not contract properly, with—Cimicif.
Thighs, from sacrum around the pubes and down the—Sulph.
" shooting down the—Lac caninum.
" extending down the anterior of—Xanthoxylum.
Toes, causing cramp in—Cuprum.
Trembling feeling all over, sense of, without trembling—*Sulph. acid.*
Twitching, with—Hyos.
Vagina, with pains shooting upward in—Sepia.
Vertigo with—Ferrum.
Violent, too—Nux-vom., Puls.
Warm room, like to have—Nux-vom.

